# Phytoene Synthase: The Key Rate-Limiting Enzyme of Carotenoid Biosynthesis in Plants

**DOI:** 10.3389/fpls.2022.884720

**Published:** 2022-04-12

**Authors:** Xuesong Zhou, Sombir Rao, Emalee Wrightstone, Tianhu Sun, Andy Cheuk Woon Lui, Ralf Welsch, Li Li

**Affiliations:** ^1^Robert W. Holley Center for Agriculture and Health, USDA-Agricultural Research Service, Cornell University, Ithaca, NY, United States; ^2^State Key Laboratory of Crop Genetics and Germplasm Enhancement, Nanjing Agricultural University, Nanjing, China; ^3^Plant Breeding and Genetics Section, School of Integrative Plant Science, Cornell University, Ithaca, NY, United States; ^4^ScreenSYS GmbH, Freiburg, Germany

**Keywords:** carotenoid, PSY, functional evolution, regulation, metabolic engineering

## Abstract

Phytoene synthase (PSY) catalyzes the first committed step in the carotenoid biosynthesis pathway and is a major rate-limiting enzyme of carotenogenesis. PSY is highly regulated by various regulators and factors to modulate carotenoid biosynthesis in response to diverse developmental and environmental cues. Because of its critical role in controlling the total amount of synthesized carotenoids, PSY has been extensively investigated and engineered in plant species. However, much remains to be learned on its multifaceted regulatory control and its catalytic efficiency for carotenoid enrichment in crops. Here, we present current knowledge on the basic biology, the functional evolution, the dynamic regulation, and the metabolic engineering of PSY. We also discuss the open questions and gaps to stimulate additional research on this most studied gene/enzyme in the carotenogenic pathway.

## Introduction

Carotenoids are a group of lipophilic isoprenoid metabolites. They play diverse roles in plants as essential photoprotective and light-harvesting pigments in photosynthesis, color agents, and precursors of phytohormones, aroma/flavor compounds, and signaling molecules. Carotenoids are also important to human nutrition and health as dietary precursors of vitamin A and antioxidants in preventing vitamin A deficiency and reducing the risk of various chronic diseases (Eggersdorfer and Wyss, 2018). The critical roles of carotenoids to plants and humans have provoked significant efforts to understand carotenoid metabolism in plants and to generate carotenoid enriched crops (Nisar et al., 2015; Giuliano, 2017; Rodriguez-Concepcion et al., 2018; Zheng et al., 2020; Sun et al., 2022).

**FIGURE 1:**
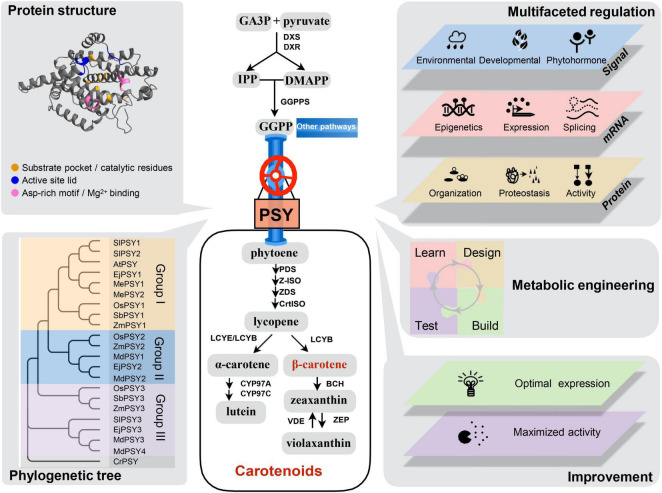
Overview of plant carotenoid biosynthesis pathway and the biology of plant phytoene synthase. Carotenoid biosynthesis starts with GA3P and pyruvate to produce the direct precursor of GGPP. PSY is the first committed and major-rate limiting enzyme in the carotenoid biosynthesis pathway. β-carotene is the most potent provitamin A carotenoid. Abbreviations for metabolites and pathway enzymes can be found in Sun et al. (2022). Protein structure: A high confident structure prediction of Arabidopsis PSY protein (https://www.alphafold.ebi.ac.uk/entry/P37271) with conserved motifs of enzymatic active site indicated. Phylogenetic tree: Land plant PSY proteins can be divided into three large groups (I, II, and III) and *PSY* genes in each group show tissue-specific expression pattern. Multifaceted regulation: PSY gene expression and activity are under multifaceted regulation by various signals and factors at epigenetic, transcriptional, post-transcriptional, and post-translational levels. Metabolic engineering: PSY is the main target gene of carotenoid metabolic engineering. The design of genetic manipulation of PSY for carotenoid enrichment benefits from the learning process. Improvement: Further understanding of the regulatory mechanisms of PSY and maximization of its activity will enable predictable engineering and breeding to achieve the targeted goals of carotenoid improvement in crops. Sl, *Solanum lycopersicum*; At, *Arabidopsis thaliana*; Ej, *Eriobotrya japonica*; Me, *Manihot esculenta*; Os, *Oryza sativa*; Sb, *Sorghum bicolor*; Zm, *Zea mays*; Md, *Malus domestica*; Cr, *Chlamydomonas reinhardtii*. The database accession numbers of sequences used are: AtPSY (AAA32836), EjPSY1 (KF922363), EjPSY2A (KF922364), EjPSY3 (KF922367), MdPSY1 (KT189149), MdPSY2 (KT189150), MdPSY3 (KT189151), MdPSY4 (KT189152), MePSY1 (ACY42666), MePSY2 (ACY42670), OsPSY1 (AAS18307), OsPSY2 (AK073290), OsPSY3 (DQ356431), SbPSY1 (AY705389), SbPSY3 (AAW28997), SlPSY1 (ABM45873), SlPSY2 (ABU40771), SlPSY3 (Solyc01g005940), ZmPSY1 (AX13806), ZmPSY2 (AAQ91837), ZmPSY3 (DQ356430), and CrPSY (Q6J214).

Carotenoids are *de novo* synthesized in nearly all kinds of plastids and are abundant in chloroplasts and chromoplasts in plant cells (Sun et al., 2018). Carotenoid biosynthesis occurs primarily in dependence of plastid membrane association and involves a group of nuclear-encoded enzymes (Shumskaya and Wurtzel, 2013). Phytoene synthase (PSY) catalyzes the first committed step of carotenogenesis by condensation of two molecules of geranylgeranyl diphosphate (GGPP) derived from the methylerythritol phosphate (MEP) pathway to produce the C40 hydrocarbon 15-*cis*-phytoene (Figure 1). Consecutive modifications of 15-*cis*-phytoene by desaturases and isomerases form all-*trans*-lycopene, which is cyclized by two cyclases to convert into either β-carotene or α-carotene. The subsequent hydroxylation and epoxidation by hydroxylases and epoxidase produce xanthophylls (Hermanns et al., 2020; Sun et al., 2020). Being a major rate-limiting and highly regulated enzyme, PSY has been extensively investigated and engineered in plant species (Burkhardt et al., 1997; Shewmaker et al., 1999; Ye et al., 2000; Ducreux et al., 2005; Paine et al., 2005; Diretto et al., 2007; Fraser et al., 2007; Maass et al., 2009; Naqvi et al., 2009; Rodriguez-Villalon et al., 2009; Welsch et al., 2010, 2018; Zhou et al., 2015; Zhu et al., 2018; Cao et al., 2019; Sun et al., 2021).

## The Basic Biology of Phytoene Synthase

The first gene encoding PSY from land plants was identified from tomato. A tomato fruit cDNA, pTOM5, which was previously shown to be highly upregulated during fruit ripening, was cloned (Slater et al., 1985). Sequencing of pTOM5 revealed homology to the bacterial crtB (Ray et al., 1987) and the pTOM5 antisense tomato plants showed strongly reduced carotenoid levels in the fruit (Bird et al., 1991). Further evidence that pTOM5 encodes a phyotene synthase came from complementation of carotenoid biosynthesis in the *crtB* mutant of *Rhodobacter capsulatus* by pTOM5, which finally confirmed its identify as fruit-specific *PSY1* (Bartley et al., 1992).

Phytoene synthase enzyme (EC 2.5.1.32) was characterized as a bifunctional enzyme, which catalyzes two tightly coupled reactions of dimerization of GGPP into prephytoene diphosphate and its subsequent conversion into phytoene (Dogbo et al., 1988). Enzymatic activity of PSY is strictly dependent on Mn^2+^ cofactor, which is believed to regulate its competition with other GGPP-consuming enzymes in plastids (Dogbo et al., 1988; Fraser et al., 2000). PSY also has a specific galactolipid and membrane association requirement for its catalytic activity (Schledz et al., 1996; Welsch et al., 2000). In general, plant PSY proteins are unstable (Mukherjee and Mukhopadhyay, 2020). This is especially true in photosynthetically active tissues, where PSY protein amounts are extremely low and subjected to a relatively high protein turnover rate. Many PSYs are known to require chaperone proteins for maintaining their stability and carotenogenic functions within plastids (Zhou et al., 2015; Park et al., 2016; Chayut et al., 2017; D’andrea et al., 2018; Welsch et al., 2018; Ahrazem et al., 2020).

Phytoene synthase belongs to the class 1 superfamily of isoprenoid biosynthetic enzymes and shares a conserved prenyltransferase domain with squalene synthase (Summers et al., 1993). PSY proteins of land plants generally contain 380–450 amino acid residues (Han et al., 2015). The active site of PSY enzymes is comprised of six conserved motifs, i.e., substrate binding pocket, catalytic residues, active lid-residues, two aspartate-rich regions, and substrate Mg^2+^ binding site (GGPP is usually complexed with Mg^2+^) (Figure 1). Most residues of the active site are identical and conserved even with the bacterial and fungal orthologs (Mukherjee and Mukhopadhyay, 2020). The three-dimensional structure modeling shows an identical structure of the active site region of PSY enzymes from evolutionarily distant lineages, despite that their global structure vary (Cao et al., 2019; Mukherjee and Mukhopadhyay, 2020).

## Functional Evolution of Phytoene Synthase

Gene duplication plays a prominent role in generating evolutionary novelty, facilitating acclimation and adaptation to adverse environments and contributing to the emergence of new agronomic traits (Conant and Wolfe, 2008; Van De Peer et al., 2017). Gene duplication events and a separate evolutionary history in land plants formed a small gene family and produced a various number of *PSY* genes in plant genomes.

In many plant species, there are two or more *PSY* paralogs belonging to three subgroups (Figure 1). Numbering of *PSY* gene nomination in tomato followed the chronological order of their identification and *PSY* paralogs identified subsequently in other plant species followed the same principle or are based on homologies to previously identified *PSY* genes. Thus, *PSY* numbering does not always expresses similarity of functional roles. Various *PSY* paralogs were recruited with overlapping functions in carotenogenesis during evolution. They acquired tissue-specific expression patterns and subfunctionalizations to fine-tune carotenoid biosynthesis in response to developmental and environmental cues (Li et al., 2008b). For instance, tomato, maize, and wheat genomes harbor three *PSY* genes, with *PSY1* primarily responsible for carotenoid accumulation in fruit or grains, *PSY2* functioning in green tissues for photosynthesis, and *PSY3* in roots to regulate ABA biosynthesis under abiotic stress or mycorrhizal symbiosis (Fraser et al., 1999; Giorio et al., 2008; Li et al., 2008a; Dibari et al., 2012; Stauder et al., 2018). In rice, both *OsPSY1* and *OsPSY2* contain light responsive *cis*-acting elements and play predominant roles in carotenogenesis in green tissues, whereas *OsPSY3* is induced in roots by high salt and/or drought stress (Welsch et al., 2008). The expression and functional evolution of *PSY* paralogs have been also observed in many other plant species. Interestingly, although Arabidopsis has experienced four gene duplication events (Bowers et al., 2003), there is only a single *PSY* gene. Perhaps the alternative splicing of *PSY* in Arabidopsis allows encoding multiple PSY isozymes to adapt to the changing environments (Alvarez et al., 2016).

While the key sites or motifs of plant PSYs are highly conserved during the enzyme evolution (Han et al., 2015; Cao et al., 2019), evolutionary processes with gene duplication, divergence, and allelic variations generated PSY enzymes with different activities among and even within the same plant species. An example is the finding that an alteration of evolutionarily conserved neighboring aromatic-aromatic amino acid combination in the PSY active site gives variable activities of PSY isoforms in tomato (Cao et al., 2019). These isoforms also evolved with different biochemical properties for the requirements of Mn^2+^ cofactor, optimal pH, and substrate affinity (Fraser et al., 2000). In cassava, a divergence of one amino acid residue in a highly conserved structure region of PSY2 altered its catalytic activity and is associated with root carotenoid content in cassava (Welsch et al., 2010). Allelic variations resulting in loss of PSY activity led to white-fleshed cultivars in loquat (Fu et al., 2014) and white petal varieties of the California poppy (Pollack et al., 2019). Moreover, allelic variation was found to give PSY isozymes distinct plastid suborganellar localization and presumably altered activity with different carotenoid sequestration structures in maize (Shumskaya et al., 2012). Uneven evolution of domains and regions of PSY was noticed in grass species (Fu et al., 2010). These examples underscore the profound impact of evolutionary processes on PSY activity and in shaping agronomic traits.

## Multifaceted Regulation of Phytoene Synthase

### Transcriptional Regulation

Phytoene synthase is a major rate-limiting enzyme in carotenoid biosynthesis and its activity effectively determines the metabolic flux to carotenoids. It is therefore not surprising that multiple mechanisms at various levels are utilized to regulate the spatiotemporal expression and activity of PSY in plants (Figure 1; Ruiz-Sola and Rodríguez-Concepción, 2012; Sun and Li, 2020; Sun et al., 2022). Transcriptional regulation is central to the control of PSY activity for carotenogenesis. Various signals and factors, such as development, phytohormone, retrograde, light, temperature, drought, salt, and circadian, as well as allelic variation, mutation, and feedback/feedforward are all known in the literature to regulate *PSY* gene expression. However, there is a large gap for the mechanistic understanding of their regulatory roles.

A number of transcription factors (TFs) were found to directly bind to the promoters of *PSY* and regulate its transcript levels in photosynthetic and non-photosynthetic tissues. During seedling de-etiolation, *PSY* transcript level is greatly induced by light (Von Lintig et al., 1997; Welsch et al., 2000). Two key photomorphogenetic regulators, Phytochrome-Interacting Factors (PIFs, repressors) and LONG HYPOCOTYL5 (HY5, activator) form a dynamic repression-activation module. They directly bind to the same G-box motif in the *PSY* promoter in dark or light to regulate *PSY* transcription in response to light, temperature, and circadian cues (Toledo-Ortiz et al., 2010, 2014). A different repression-activation module involving PIF1 and Phytochrome Rapidly Regulated 1 (PAR1) is recruited to regulate *PSY* expression in response to shade (Bou-Torrent et al., 2015). In addition, RAP2.2 binds to the ATCTA element in the *PSY* promoter with modest regulatory activity, suggesting RAP2.2 being one element of the complex regulatory network (Welsch et al., 2007).

Many fruit-specific *PSYs* are transcriptionally upregulated during fruit ripening (Lu P. et al., 2018). Several TFs including Ripening Inhibitor (RIN), Tomato AGAMOUS-LIKE1 (TAGL1), FRUITFULL1 (FUL1), *Sl*BBX20, *Sl*WRKY, *Sl*MYB72, and *Sl*PIF1 directly bind to the *SlPSY1* promoter to positively or negatively regulate its expression in tomato fruit (Itkin et al., 2009; Vrebalov et al., 2009; Fujisawa et al., 2013, 2014; Llorente et al., 2016; Xiong et al., 2019; Wu et al., 2020). In citrus, both *Cs*MADS5 and *Cs*MADS6 bind and regulate *CsPSY* transcription and these two TFs physically interact with each other, possibly forming an enhancer complex to promote carotenogenic activity (Lu S. et al., 2018; Lu et al., 2021). While multiple TFs are shown to directly activate *PSY* gene expression in non-photosynthetic tissues, it remains unclear whether they are the primary regulators, function across plant species, form regulatory modules with others, and/or represent *bona fide* regulators of *PSY*.

Modulation of *PSY* expression has also been documented by other mechanisms. Uncharacterized carotenoids or degraded products were found to negatively regulate *SlPSY1* in tomato or PSY protein level in carrot root, constituting a negative feedback regulation of PSY activity (Kachanovsky et al., 2012; Arango et al., 2014; Enfissi et al., 2017). Perturbation of carotenoid biosynthesis has also been observed to activate PSY2 activity in tomato and pepper fruits when PSY1 is not functional (Jang et al., 2020; Gupta et al., 2022; Karniel et al., 2022). Epigenetic regulation such as histone modification as well as DNA methylation and demethylation modifies *SlPSY1* mRNA levels during tomato fruit ripening (Liang et al., 2017; Liu et al., 2020; Sun et al., 2022).

### Post-transcriptional and Post-translational Regulation

Additionally, PSY expression and enzymatic activity are also regulated at post-transcriptional and post-translational levels (Figure 1). Mechanisms comprising of alternative splicing, protein-protein interactions, and multi-enzyme complexes constitute efficient, rapid, and dynamic regulation to fine-tune PSY activity and carotenogenesis (Ruiz-Sola and Rodríguez-Concepción, 2012; Nisar et al., 2015; Sun and Li, 2020; Sun et al., 2022).

Alternative splicing changes *PSY* transcript sequence length, producing variants with different translation efficiency and/or distinct enzyme activity to control the functional PSY in Arabidopsis leaves (Alvarez et al., 2016), tomato fruit (Chen et al., 2019), saffron stigmata (Ahrazem et al., 2019), and bread wheat endosperm (Howitt et al., 2009). Such post-transcriptional regulatory mechanisms may also provide an alternative toward employing multiple copies of a gene. Moreover, regulation of translational activity *via* the 5’UTR of *PSY* transcripts might represent a regulatory mode that allows a fast adaptation of PSY protein abundance and thus biosynthetic activity toward carotenoid requirement. Considering that PSY translation takes place in the cytoplasm while (lipophilic) carotenoids are present in plastids, an attractive hypothesis includes shuttling of regulatory, hydrophilic carotenoid degradation products *via* plastid membranes (Alvarez et al., 2016).

Protein-protein interaction is fundamentally important to maintain and fine-tune metabolic processes in plant cells (Struk et al., 2019). The interactions of PSY protein with OR chaperones and Clp proteases exemplify the crucial role of protein-protein interactions in regulating carotenogenic enzyme activity, proteostasis, and carotenoid biosynthesis. PSY physically interacts with OR proteins to maintain its activity through OR chaperone activity (Yuan et al., 2015; Zhou et al., 2015; Park et al., 2016; Chayut et al., 2017; Welsch et al., 2020). PSY directly interacts with Clp protease to mediate its degradation (D’andrea et al., 2018; Welsch et al., 2018). Through the protein-protein interactions, OR and Clp proteins counterbalance each other to adjust the functional form and proteostasis of PSY in plant cells. In addition, PSY is associated with GGPPS to facilitate channeling of precursor for carotenoid biosynthesis in Arabidopsis, pepper and tomato (Ruiz-Sola et al., 2016; Wang et al., 2018; Barja et al., 2021) SGR was found to physically interact with CsPSY protein to regulate carotenogenesis in citrus (Zhu et al., 2021). Post-translational modification *via* ubiquitination-mediated turnover of non-imported PSY precursors outside of plastids was noted *via* direct interaction between SlPSY1 and a E3 ubiquitin ligase (Wang et al., 2020).

Enzyme complexes and metabolons facilitate metabolic flux. As lipophilic carotenoids are synthesized from small hydrophilic precursors (IPP, DMAPP), but include amphiphilic intermediates like GGPP, free substrate diffusion can be excluded and metabolite channeling between different enzymes of the pathway plays an essential role. Importantly, the PSY substrate GGPP is shared with other pathways (Figure 1), such as chlorophyll and tocopherol biosynthesis in chloroplasts. However, there are mainly one or two GGPPS isoenzymes that serve all GGPP-consuming pathways (Ruiz-Sola et al., 2016; Barja et al., 2021). This situation raises the question whether competitive interaction between enzymes of off-branching pathways regulates flux into various pathways. Interestingly, a synthetic GGPPS-PSY metabolon has been demonstrated with increased efficiency in channelizing GGPP into carotenogenesis (Camagna et al., 2019). An early study also suggests that PSY is associated with a large enzyme complex containing IPI and GGPPS in chloroplasts of tomato plants for active carotenogenesis (Fraser et al., 2000).

## Genetic Engineering of Phytoene Synthase

Since carotenoids, as important nutrients in human diets, are deficient or low in many food crops, enormous endeavors have been made to engineer crops with enriched carotenoid content (Giuliano, 2017; Zheng et al., 2020). The learning process of multifaceted regulation provides constitutive input to design, build, and test metabolic engineering module of carotenoids (Figure 1). *PSY* is the main target for manipulation as it is the major rate-limiting and key enzyme driving the flux into carotenogenesis. Because *PSY* genes are not expressed in rice endosperm in all rice germplasms, the use of genetic engineering to obtain the capacity for carotenoid biosynthesis is required for Golden Rice (Beyer et al., 2002). Endosperm specific expression of a highly efficient *ZmPSY* along with a bacterial phytoene desaturase (*crtI*) generated β-carotene enriched Golden Rice (Paine et al., 2005) and CRISPR golden rice (Dong et al., 2020), as well as orange maize (Naqvi et al., 2009) and astaxanthin rice with two additional genes (Zhu et al., 2018). Tissue-specific expression of plant or bacteria *PSY* also dramatically increases α-carotene and β-carotene in many tissues such as wheat grain (Wang et al., 2014), canola seeds (Shewmaker et al., 1999), cotton seed (Yao et al., 2018), soybean seed (Schmidt et al., 2015), eggplant fruit (Mishiba et al., 2020), Arabidopsis seed (Lindgren et al., 2003; Sun et al., 2021), and potato tuber (Ducreux et al., 2005). Constitutive expression of *PSY* either under CaMV35S or other promoters such as maize polyubiquitin shows enhanced carotenoid levels in tomato fruit (Fray et al., 1995; Fraser et al., 2007), banana (Paul et al., 2017), cassava root (Welsch et al., 2010), and carrot root (Maass et al., 2009), although undesirable or pleiotropic phenotypes by constitutive *PSY* expression are observed in some cases.

Manipulation of *PSY* with or without other carotenogenic genes often results in increased carotenoid levels. However, the synthesized carotenoids are subjected to oxidative degradation, particularly during grain seed maturation and post-harvest storage such as in Golden Rice, sorghum, and maize (Farre et al., 2013; Che et al., 2016; Schaub et al., 2017). Co-expression of *PSY1* with barley homogentisate geranylgeranyl transferase (*HGGT*) to increase antioxidant vitamin E provides a strategy to improve β-carotene content and stability as shown in sorghum grain (Che et al., 2016). In addition, co-expression of *PSY* with *OR^His^*, a nature variant of *OR* that induces chromoplast biogenesis, offers an additional strategy to significantly boost carotenoid accumulation and stability during seed maturation and storage as documented in Arabidopsis seed (Sun et al., 2021). Expression of an *OR* mutant variant has also shown to enhance carotenoid levels and stability in potato and sweetpotato (Lu et al., 2006; Li et al., 2012; Kim et al., 2021). No other regulators were tested for carotenoid enrichments in starch-rich organs despite of their reported roles in mediating carotenogenesis.

The intrinsic properties of PSY determine its efficiency. Numerous examples exemplify a simple change in PSY amino acid sequences that profoundly alters its activity (Welsch et al., 2010; Shumskaya et al., 2012; Cao et al., 2019), which provides the potential to rationally design PSY with high enzyme activity. Various approaches are available to facilitate rapid screening of PSY variants with improved efficiency, such as *via* heterologous expression in bacterial test system and in callus system, which seems transferable to various plant systems (Paine et al., 2005; Bai et al., 2014; Schaub et al., 2018; Camagna et al., 2019; Cao et al., 2019; Camagna and Welsch, 2020). Application of improved PSY variants is expected to effectively develop carotenoid enriched crops.

PSY is known to be the key rate-limiting enzyme of carotenogenesis. However, the availability of precursors and products following altering pathway enzyme expression and activities can create new metabolic bottlenecks in the pathway (Bai et al., 2016, 2017). Indeed, metabolic control analysis reveals that the flux control coefficient of PSY1 is predominant in the wild type fruit but reduces in the PSY transgenic tomato, and consequently the flux control is likely shared by several enzymes in the carotenoid pathway (Rios-Estepa and Lange, 2007). Thus, effective engineering of carotenoid metabolic pathway may benefit from multistep manipulation in some cases.

## Conclusion and Future Research

Because of its critical role in carotenogenesis, PSY is the most well characterized and studied gene/enzyme in the carotenoid biosynthesis pathway. The accelerated sequencing of numerous plant genomes and transcriptomes makes it straightforward to identify *PSY* gene family members and to reveal their tissue-specific expression patterns in plant species. Extensive studies of PSY in carotenogenesis have also established various regulatory tiers acting at transcriptional, post-transcriptional, post-translational, and epigenetic levels. At the same time, great success has been made in engineering *PSY* expression to enhance crop nutritional quality. However, considerably more efforts are still required to delineate the mechanistic details of various regulatory controls and to develop/select highly efficient PSY enzyme (Figure 1). Open questions and gaps for the most studied enzyme in the pathway remain, including the followings.

What are the additional regulators that govern PSY gene expression, protein stability, and activity? The crucial role of PSY in carotenogenesis and multiple signals/factors affecting its expression suggest a complex regulatory control machinery and network. Although a number of TFs have been shown to bind directly to the *cis*-motifs in the promoters of *PSY*, additional regulators that may have dominant or additive roles in transcriptionally regulating *PSY* remain uncovered. In addition to a few post-translational regulators identified, many more are expected to be identified for rapid regulation of PSY activity and fine-tune of carotenogenesis.

What are the key amino acid residues that give high PSY activity? The intrinsic enzymatic efficiency of PSY enzymes from various plant species varies greatly to give different biosynthetic capacity as exemplified with daffodil and maize PSY used for Golden Rice 1 and 2, respectively. Single amino acid sequence changes can profoundly alter PSY activity. Therefore, identification and optimization of the key amino acid residues will not only unravel the intrinsic features of PSY activity, but also facilitate the design of highly efficient PSY for development of carotenoid enriched crops.

How does PSY work with other enzymes in the pathway to drive the metabolic flux and what are the mechanisms controlling metabolon assembly? It has long been hypothesized that carotenogenic enzymes form enzyme complexes although solid evidence is still lacking. GGPS-PSY metabolon can efficiently drive GGPP substrate into the carotenogenic pathway, supporting an important role of the complexes for carotenogenesis.

One interesting phenomenon observed in many studies is that overexpression of *PSY* is often associated with increased β-carotene accumulation in many non-photosynthetic tissues or organs. What are the regulatory mechanisms for this favorable phenomenon? Identification of the mechanisms involved might further help to develop additional approaches for crop nutritional quality improvement.

Phytoene synthase activity needs to be precisely coordinated with carotenoid end products. Of special interest for future research also includes how the rate-limiting function of PSY is dynamically regulated with the total carotenoid content and the relative levels of individual carotenoids.

The fast advance in techniques and multi-omics information from many plant species will be promising in elucidating the unknown regulators of PSY and unraveling the intrinsic features underlying its activity and function. The information obtained would greatly enrich our current knowledge of the regulatory mechanisms and lay down novel strategies for predictable carotenoid improvement in crops. Integrating both key regulators and targeted carotenogenic genes into a synthetic biology framework will allow us to achieve the targeted biofortification goals.

## Author Contributions

XZ wrote the Sections “Functional Evolution of Phytoene Synthase” and “Genetic Engineering of Phytoene Synthase” as well as formatted the references. SR wrote the Sections “Basic Biology of Phytoene Synthase” and “Multifaceted Regulation of Phytoene Synthase.” EW wrote the Section “Introduction.” TS designed Figure 1. TS, AL, and RW critically revised the manuscript. LL prepared the final version with input of all authors. All authors contributed to the article and approved the submitted version.

## Conflict of Interest

RW was employed by ScreenSYS GmbH. The remaining authors declare that the research was conducted in the absence of any commercial or financial relationships that could be construed as a potential conflict of interest.

## Publisher’s Note

All claims expressed in this article are solely those of the authors and do not necessarily represent those of their affiliated organizations, or those of the publisher, the editors and the reviewers. Any product that may be evaluated in this article, or claim that may be made by its manufacturer, is not guaranteed or endorsed by the publisher.
